# Remedies and Their Uses

**Published:** 1906-08-04

**Authors:** 


### Remedies and their Uses.

### Substitutes for Iodoform.

Iodol.—This substance is a derivative of pyrrol, which has the formula:—

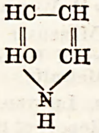

by substituting the four hydrogen atoms combined with carbon by iodine, thus:—

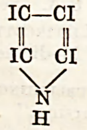

It is a fine powder of a pale yellow or brownish colour. It is tasteless, odourless, and slightly greasy to touch. It is easily soluble in absolute alcohol and ether; but solutions in alcohol cannot be diluted with water, as iodol is but slightly soluble in that substance. The solutions must not be subjected to greater heat than 40° C. Iodol contains 89 per cent. of separable iodine, and, unlike iodoform, this iodine is split off by healthy body fluids, and not only in presence of purulent material. It is said not to irritate the skin like iodoform, and it does not cake. Its toxicity, compared with iodoform, is as 2 to 3, and it is slowly eliminated, mainly by the kidneys. Speaking generally, iodol has been employed for all cases when iodoform would be considered indicated—for granulating wounds, specific and non-specific ulcers, and as an antiseptic and deodorant for foul discharging surfaces. It can also be used for plugging bone cavities, and in ear, nose, and eye work. For steeping wound dressings or plugs, Mazzoni's solution in glycerine is recommended, namely:—

| | | |
|---|---|---|
| ℞ | Iodol ... ... ... ... ... ... | ʒj. |
| | Sp. vini rect. ... ... ... ... ... | ʒiv. |
| | Glycerini ... ... ... ... ... ... | ʒx. |

The iodol is dissolved in the spirit by the aid of gentle heat, and the glycerine then added. It should be kept in a yellow glass bottle.

Ointments may be prepared with a basis of vaseline or lanoline in strengths of gr. v. to ʒss. to the ounce, and a collodion containing gr. xxiv. to the half-ounce is a convenient form for application. To prepare an emulsion similar to that of iodoform, so often used for tuberculous and other cavities, it is only necessary to use water as a diluent.

| | | |
|---|---|---|
| ℞ | Iodol ... ... ... ... ... ... | ʒj. |
| | Alcohol absol. ... ... ... ... ... | ʒj. |
| | Glycerini ... ... ... ... ... ... | ʒij. |
| | Aquæ dest. ... ... ... ... ... ... | ʒij. |

The water is gradually added to the other ingredients. Iodol is manufactured by Kolle and Co., Biebrich-am-Rhein.

Europhen.—This compound (isobutyl orthocresoliodide liberates iodine. It is a yellow powder insoluble in water, but soluble in water and fixed oils. It contains 28.1 per cent. iodine. Its lightness gives it a great "covering" capacity as a dusting powder. It may, like iodoform, be used with an equal part of boracic acid, or in the form of a 10 per cent. ointment. It is odourless, and is said to be non-toxic; hard and soft venereal ulcers have been successfully treated with this preparation. Europhen gives off small amounts of iodine at ordinary temperatures in contact with moisture, and when heated to 70° C. an energetic liberation of the element takes place. Alkalies (such as the tissue fluids) facilitate the decomposition, so that it may be regarded as a rapidly acting substance. Hence the usual dilution with boric acid. A collodion (20 per cent. europhen in collodion flexile) is a useful dressing for cuts and minor surgical operations. For eczema intertrigo, Saalfeld has used a fatty powder containing—

| | |
|---|---|
| Europhen ... ... ... ... ... ... | 5 |
| Lanoline ... ... ... ... ... ... | 5 |
| Talc powder ... ... ... ... ... ... | 90 |

The preparation is made by Bayer and Co., of Elberfeld.

